# Natural Ingredients of Transdermal Drug Delivery Systems as Permeation Enhancers of Active Substances through the *Stratum Corneum*

**DOI:** 10.1021/acs.molpharmaceut.3c00126

**Published:** 2023-06-06

**Authors:** Natalia Schafer, Radosław Balwierz, Paweł Biernat, Wioletta Ochędzan-Siodłak, Jacek Lipok

**Affiliations:** † Faculty of Chemistry, University of Opole, 45-052 Opole, Poland; ‡ Department of Drug Forms Technology, Faculty of Pharmacy, Wroclaw Medical University, 50-556 Wroclaw, Poland

**Keywords:** penetration enhancers, raw materials, skin barrier, terpenes, fatty acids, polysaccharides

## Abstract

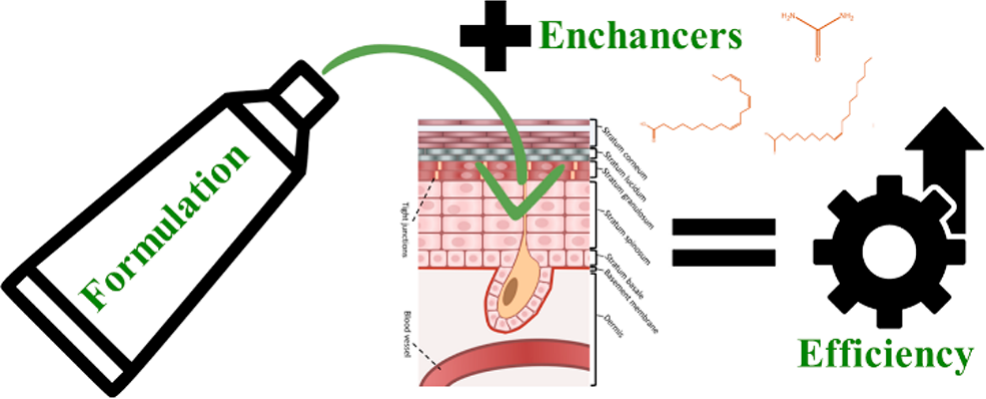

In recent years, significant progress has been made in transdermal drug delivery systems, but there is still a search for enhancers that can improve the absorption of active substances through the *stratum corneum*. Although permeation enhancers have been described in the scientific literature, the use of naturally occurring substances in this role is still of particular interest, because they can offer a high level of safety of use, with a low risk of skin irritation, and high efficiency. In addition, these ingredients are biodegradable, easily available, and widely accepted by consumers due to the growing trust in natural compounds. This article provides information on the role of naturally derived compounds in transdermal drug delivery systems that help them penetrate the skin. The work focuses on the components found in the *stratum corneum* such as sterols, ceramides, oleic acid, and urea. Penetration enhancers found in nature, mainly in plants, such as terpenes, polysaccharides, and fatty acids have also been described. The mechanism of action of permeation enhancers in the *stratum corneum* is discussed, and information on the methods of assessing their penetration efficiency is provided. Our review mainly covers original papers from 2017 to 2022, supplemented with review papers, and then older publications used to supplement or verify the data. The use of natural penetration enhancers has been shown to increase the transport of active ingredients through the *stratum corneum* and can compete with synthetic counterparts.

## 1 Introduction

Transdermal drug delivery (TDD) systems are one of the most widely researched pharmaceutical products.^1^ TDD is a convenient alternative to intravenous, intramuscular, and oral routes of administration. The transdermal route avoids the first-pass effect in the liver making this method suitable for drugs that have low bioavailability when administered through the oral route or exhibit adverse effects due to biotransformation.^2^ This method also has the advantage of being painless, noninvasive, and easy to apply and having controlled release (modified release) which can prolong the therapeutic effect.^3^ Nevertheless, the skin acts as an external barrier to prevent exogenous compounds from entering the body, including drugs.^4^ This presents a significant challenge for TDD researchers in the development of methods to penetrate the top layer of the epidermis: the *stratum corneum* (SC).^5^ The nonpolar, hydrophobic nature of the SC and the physiological property of being permeated by only those particles whose atomic weight does not exceed 500 Da substances makes polar, hydrophilic particles cross this barrier rarely or not at all.^6^ An intact SC is crucial in maintaining legitimate skin function. Its dysfunctions are seen during dermatological diseases, such as atopic dermatitis or ichthyosis.^5^ Transdermal drug delivery systems should be applied to healthy skin, so methods are being sought that will temporarily interact with the components of the epidermis, temporarily changing its permeability to therapeutic substances, at the same time not changing the activity of the active substance, not causing its degradation, and not causing skin irritation and permanent skin damage.^7^

The key to producing an effective transdermal system is the right choice of the type of formulation. The degree of drug diffusion through the skin is influenced by the type of TDD formulation, i.e., ointment, cream, gel, or transdermal patch.^8,9^ These formulations include different types of gels (hydrogels, organogels, bigels, emulgels, and nano gels), emulsions, (microemulsions, nanoemulsions, and multiple emulsions), and liquid crystals, which are intermediate between the solid and liquid form.^10−12^ Furthermore, the development of nanotechnology has enabled the incorporation of APIs into carriers such as liposomes, niosomes, nanostructured lipid carriers, solid lipid nanoparticles, polymer nanoparticles, micelles, dendrimers, carbon nanotubes, etc.^13,14^

Moreover, active and passive methods are used to facilitate TDD. Active methods involve the use of external energy to increase the penetration of active pharmaceutical ingredients (APIs) through the skin by using electrical energy (iontophoresis, electroporation), ultrasound (sonophoresis), radiofrequency electromagnetic waves (radiofrequency), and laser energy.^15−20^ These methods include mechanical techniques such as micropuncture to create pores in the SC for APIs.^21−24^ Passive methods involve the interactions between the drug, vehicle, and SC layer. They aim to modify the API physicochemical properties, e.g., by changing its solubility or ionization and/or leading to an increase in skin permeability. Given this, eutectic systems, prodrugs, ion-pair technique, and supersaturated systems are used. Developments in pharmaceutical and chemical sciences have now made it possible to use chemical compounds that, by interacting with SC components, improved the permeability of drugs.^25,26^

The eutectic system is a mixture of two or more substances formed by entropy changes associated with both hydrogen bonds and van der Waals forces. These interactions result in a mutual decrease in the melting point of the mixture with respect to each of its individual components.^11,27,28^ The pro-drug technique is based on the modification of a substance’s active molecules by attaching a lipophilic group, such as an ester group, to create a compound that is more easily partitioned between the SC and the pharmaceutical formulation causing a change in the partition coefficient (LogP).^29−32^ The so-called ion-pair technique which involves neutralization of the electrical charge of the active substance by through formation its salt is also used.^33−35^ Supersaturated systems allow for faster transdermal penetration of the drug, as they have increased thermodynamic activity.^36^

Among chemical compounds that increase the permeability of the skin to drugs are those otherwise known as chemical penetration enhancers (CPEs) or sorption promoters: terpenes, terpenoids, sulfoxides, laurocapram (Azone), pyrrolidones, fatty acids, fatty alcohols, alcohols containing glycols, urea, and surfactants.^25,26^ In 2021, 649 compounds classified as CPEs were collected in a database which included the following groups of chemicals: alcohols and polyols, lactams and their analogues (azepane, azone, caprolactam, morpholine, piperazine, piperidine, piperidone, pyrrolidine, pyrrolidone, and succinimide), esters and ethers, fatty acids, terpenes and steroids, and miscellaneous additives such as amino acids, aliphatic compounds, aromatic compounds, and inorganic compounds.^37^ However, some of the listed groups of compounds, primarily morpholine and morpholine derivatives and among them Azone,^38^ surfactants,^39^ aromatic compounds,^40^ and many others,^38^ carry the risk of causing skin irritation, permanent disorganization of the skin barrier, and toxic effects on skin cells. These effects may not be acceptable in the application of transdermal drug delivery systems to the skin. High potential for safe use and a low risk of skin irritation is presented by transdermal formulations based on substances of natural origin. In addition, these ingredients are biodegradable, readily available, and widely accepted by consumers due to growing reliance on natural occurring compounds.^41,42^ These advantages make them commonly used in cosmetic and pharmaceutical formulations. They are increasingly used as substrates for the application of active ingredients^43,44^ and as well as compounds that facilitate the penetration of other substances, including APIs.^45,46^

The most popular permeation enhancers that occur in SC are sterols, ceramides, fatty acids (oleic acid), and urea. The second group of natural enhancers that occur in nature, mainly in plants, are fatty acids and terpenes. The main sources of fatty acids are plant oils, while terpenes and terpenoids are the main components of essential oils.^47−49^

Plant oils possess skin barrier restoration and regenerative features, as well as antioxidant, and anti-inflammatory properties.^50^ They are valuable ingredients in pharmaceutical and cosmetic products, where they act as both active substances^43,44^ and compounds that facilitate the penetration of other substances, including medications.^45,46^ They are considered nontoxic and safe for topical use. Some divergence has been observed in the effects of plant oils on the skin. While sunflower seed oil contributes to improving the hydrolipidic layer of the skin, olive oil negatively affects the integrity of SC components.^51^ It has been suggested that these properties depend on the ratio of oleic to linoleic acid in the oil composition, as only the former contributes to an increase in SC permeability, facilitating the penetration of the therapeutic substance through the skin.^44,52,53^ Not without significance is the content of the unsaponifiable fraction in oils, which include compounds such as triterpene alcohols, squalene, phytosterols, flavonoids, and phospholipids, which may also potentially affect the barrier properties of the epidermis.^44^

Essential oils contain medicinal properties such as antiseptic, antiparasitic, antiviral, antifungal, and antibacterial activities.^54^ However, essential oils carry a risk of skin irritation if used undiluted or in too high of a concentration. In topical preparations, essential oils are used in concentrations between 0.5–5% and sometimes up to 10%, depending on the specific oil.^55^

It should be noticed that by using them in pharmaceutical and cosmetic products for external application as a base and/or active substance their disorganizing effect on the skin is overlooked. Consequently, a given preparation may have unforeseen and undesired effects on the skin, such as increased transepidermal water loss and skin inflammation. When used in daily skin care, these compounds can have the above-mentioned adverse effects, but on the other hand, they can be a component of the transdermal therapeutic systems, using their disorganizing effect on the protective barrier to facilitate the penetration of active substances. Therefore, it was deemed necessary to report the role of these natural compounds used in systems that facilitate the penetration of APIs through the skin.

This article focuses on the role of individual substances in enhancing the penetration of APIs. The use of natural substances as penetration enhancers of active substances in TDD systems is in line with the trend of using naturally occurring raw materials in pharmaceutical and cosmetic formulations.^41,42^ Their popularity is due to their easy availability. In addition, these compounds are considered environmentally safe and biodegradable.

The mechanism of action of natural penetration enhancers is important in determining the safety of the different ingredients. This information is particularly important for pharmaceutical and cosmetic manufacturers, as it helps in the selection of ingredients derived from natural raw materials to be used in transdermal systems. This paper provides information on which components should be avoided when a restorative effect on the epidermal barrier is needed. This is important in the pathogenesis of many dermatological defects.

Our literature review demonstrates the relationship between natural substances used in TDD systems and their effects on the SC in terms of functional changes, as well as highlighting the analytical methods used to assess these changes. The electronic databases such as Scopus, PubMed, and Medline formed the basis of the information search.

The databases were searched from March 15, 2022, to July 1, 2022, with papers from 2017–2022 considered first, followed by older publications to supplement or verify the data. Information was supplemented in the period from March 1 to March 15, 2023. Only publications in English were included. Our review consisted of mainly original papers that were supplemented with review papers. The search method for scientific articles consisted of entering keywords ranging from general ones such as TDD systems, chemical penetration enhancers, natural chemical penetration enhancers, SC, skin barrier, and biological barriers, to more specific ones such as ceramides, sterols (especially cholesterol and cholesterol sulfate), fatty acids, terpenes, urea, vegetable oils, essential oils, and polysaccharides, linking them together with AND and OR logical connectors. These substances were chosen because they naturally occur in the SC or are commonly present as active ingredients in plants. The safety of their use is well-defined, but information on their use and benefits is not obvious. The present article gives information on the concentrations of the applied promoters, the APIs that can be used with natural enhancers, and the effects that can be obtained by using the APIs together with the absorption promoters (Table 1).

**Table 1:** Efficacy of Applying Natural Permeation Enhancers with the Active Substances Used in TDDs

no.	API	isolated permeation enhancer or oil from a natural orign	substances naturally present in the skin	permeation enhancer in drug formulations	research type	research model	research technique	concentration of the enhancer	efficiency	refs
1	theophylline		CER NS (C6)		*in vitro*	model lipid membranes	Franz diffusion cells		a	(56)
	indomethacin									
2	ethyl-*p*-aminobenzoate		CER NS (C16)		*in vitro*	model lipid membranes	PermeGear in-line diffusion cells		a	(57)
3	interferon alfa-2B		oleic acid		*in vitro*	full thickness human breast skin	in-line Bronaugh flow-through diffusion cells	10%	c	(58)
4	urea		oleic acid		*in vitro*	model lipid membranes	Franz-type diffusion cells	10%	b	(59)
5	caffeine									
6	diclofenac sodium									
7	insulin		oleic acid		*in vitro*	rat skin	Franz-type diffusion cells	4.2 μL	b	(60)
8	insulin		linolenic acid						c	
9	caffeine		urea		*in vitro*	porcine skin	Franz-type diffusion cells	10%	a	(61)
10	haloperidol	limonene			*in vitro*	epidermal membranes from abdomal human skin	Franz-type diffusion cells	5%	a	(62)
11	ligustrazine hydrochloride	menthol			*in vitro*	porcine skin	Franz-type diffusion cells	3%	a	(63)
12	indometacin	camphor			*in vitro*	rat skin	TK-20B diffusion apparatus	3%	a	(64)
13	lidocaine									
14	aspirin									
15	antipyrine									
16	Tegafur									
17	5-fluorouracil									
18	osthole	borneol			*in vitro*	rat skin	Franz-type diffusion cells	1.2%	a	(65)
19	theophyline	6-(dimetyloamino)heksanian cytronellyl			*in vitro*	epidermal membranes from abdomal human skin	Franz-type diffusion cells	30 mM concentration	a	(66)
20	hydrokortyzon									
21	naproxen sodium	*Lavandula angustifoli* oil			*in vitro*	rat skin	Franz-type diffusion cells	0.5%	a	(67)
22	ibuprofen	*Radix angelicae sinensis* oil			*in vitro*	rat skin	Franz-type diffusion cells	3%	a	(68)
		*Rhizoma chuanxiong* oil								
		*Rhizoma cyperi* oil								
		*Cinnamomum cassia* oil								
		*Flos caryophylli* oil								
23	5-fluorouracil	*Sinapis alba* oil			*in vitro*	rat skin	Franz-type diffusion cells	5%	a	(69)
24	Paeonol									
25	Osthol									
26	ibuprofen	*Epilobium angustifolium* oil			*in vitro*	porcine skin	Franz-type diffusion cells		a	(70)
27	lidocaine									
28	caffeine									
29	donepezil hydrochloride	*Ledum palustre* oil			*in vitro*	rat skin	horizontal diffusion cells	10%	a	(71)
30	dihydroquercetin	soybean oil			*in vitro*	abdomal human skin	Bronaugh-type flow-through diffusion cells	0.5%	a	(72)
		olive oil								
31	flurbiprofen	olive oil			*in vitro*	abdomal human skin	Franz-type diffusion cells	20%	a	(73)
32	diltiazem hydrochloride	*Mesua ferrea* oil			*ex vivo*	porcine skin	Keshary–Chien glass	15%	a	(74)
33	*trans*-resveratrol	*Punica granatum* oil			*in vitro*	porcine skin	Franz-type diffusion cells	2.5–10%	a	(75)
34	caffeine	*Hibiscus rosa-sinensis L.* leaves mucilage			*in vitro*	rat skin	vertical diffusion cells	2%	a	(76)
35	minoxidil			nanoemulsion with oleic acid or eucalyptol	*in vitro*	epidermal membranes from abdomal human skin	Franz-type diffusion cells	14.63–15.93%	a	(77)
36	caffeine			nanoemulsion with oleic acid or eucalyptol	*in vitro*	epidermal membranes from abdomal human skin	Franz-type diffusion cells	14.63–15.93%	a	(78)
37	naproxen									
38	caffeic acid from propolis (*Apis trigona*)			nanoemulgel with oleic acid	*in vitro*	rat skin	Franz-type diffusion cells	1.25–2.5%	a	(79)
39	2,3,5,4′-tetrahydroxystilbene 2-O-β-d-glucoside			vesicles with oleic acid	*in vitro*	porcine skin	TP-6 Franz diffusion cell	32 μL	a	(80)
40	recombinant human growth hormone			complex urea/hydroxy propyl-beta cyclodextrin	*in vitro*	rat skin	Franz-type diffusion cells	12.1%	a	(81)
41	methotrexate			nanoemulsion containing chaulmoogra oil	*in vitro*	rabbits skin	Franz-type diffusion cells	84.54 μg/mL	a	(82)
42	diltiazem hydrochloride			*Ficus reticulata L*. fruit mucilage as a matrix in transdermal patches	*in vitro*	rat skin	Keshary–Chien diffusion cell	5–25%	a	(83)
43	diclofenac sodium			*Ficus carica L*. fruit mucilage as a matrix in transdermal patches	*in vitro*	rat skin	Keshary–Chien diffusion cell	4–20%	a	(84)
44	diltiazem hydrochloride			*complex Colocasia esculenta* (taro) corms *mucilage/*hydroxypropylmethylcellulose as a matrix in transdermal patches	*in vitro*	dialysis cellulose membrane	Franz-type diffusion cells	0.5–2%	a	(85)
45	isoliquiritigenin			hydrogels containing complex hyaluronic acid/hydroxyethyl cellulose	*in vitro*	rat skin	Franz-type diffusion cells		a	(86)
46	luteolin			hydrogels containing complex hyaluronic acid/poly(*N*-isopropylacrylamide)	*in vitro*	micropig dorsal skin	Franz-type diffusion cells		a	(87)
47	bovine serum albumin			hydrogels with hyaluronic acid	*in vitro*	pig skin	Franz-type diffusion cells	5%	a	(88)
48	ketoprofen			liposomes with hyaluronic acid	*ex vivo*	pig skin	Franz-type diffusion cells	1%	a	(89)
49	rhodamine B			ethosomes with hyaluronic acid	*in vitro*	rat skin	Franz-type diffusion cells		a	(64)
50	curcumin			ethosomes with hyaluronic acid	*in vitro*	mouse skin	vertical diffusion cells		a	(90)
51	tacrolimus			polymeric nanoparticles with hyaluronic acid	*ex vivo*	mouce skin	Franz-type diffusion cells	0.1–0.5%	a	(91)
52	betamethasone valerate			polymeric nanoparticles with hyaluronic acid	*ex vivo*	rat skin	Franz-type diffusion cells	1–6 mg/mL	a	(92)

a Improved drug penetration.

b There was no significant change in the drug’s permeation.

c Reduced drug penetration.

## 2 Characterization of the Epidermal Barrier in Terms of APIs Delivered by the Transdermal Route

Studies evaluating the permeation efficacy of substances applied to the skin surface for potential use in TDDs most often refer to the SC region. Research aimed at understanding the mechanism of action of permeation enhancers at a molecular level is particularly important. For this purpose, a brief description of the structure of the SC is necessary.

**Figure 1:**
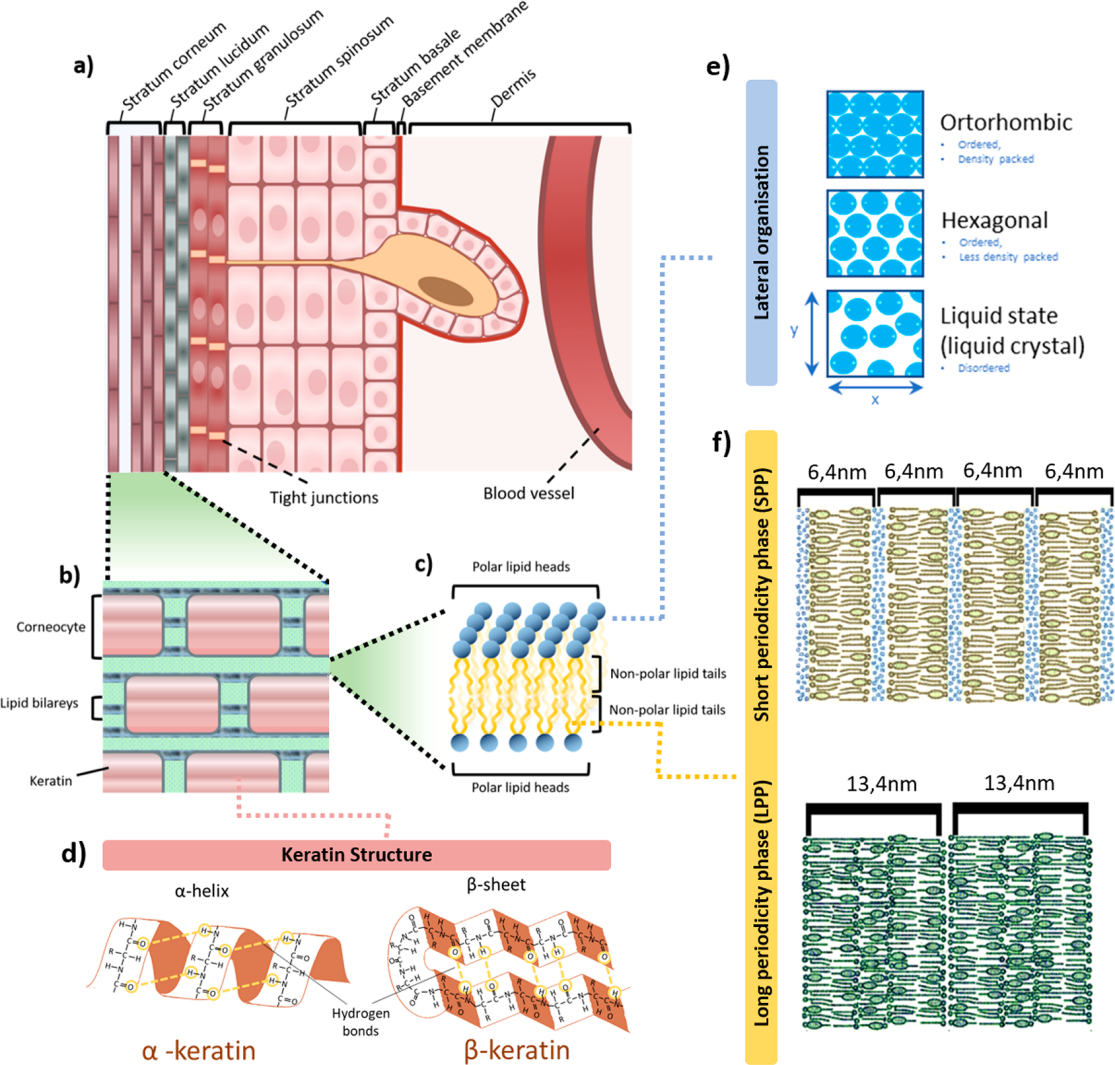
Skin structure. (a) Structure of the epidermis. (b) Schematic diagram of the structure of the SC. (c) Schematic diagram of the arrangement of lamellar lipids. (d) Schematic structure of keratin. (e) Three packing forms of the barrier lipids: orthorhombic, hexagonal, or liquid state (liquid crystal) arrangement. (f) Repeated phases of lamellar lipids: LPP and SPP with a water layer (highlighted in blue) between the polar lipid heads.

The SC is 10–20 μm thick and consists of 15–20 layers of flattened, densely packed keratin-filled corneocytes separated by a lamellar intercellular lipid system (Figure 1a,b). The remainder of the epidermis is 50–120 μm thick of stratified squamous epithelium in which no blood vessels are present. It consists of a *lamina propria*, granular layer, squamous layer, and basement membrane separating the epidermis from the dermis.^93^

Small-angle X-ray scattering data obtained by Bouwsta et al. indicates that the SC lamellar lipid system can be divided into two recurring, characteristic phases, namely, a 6.4 nm short periodicity phase (SPP) and a 13.4 nm long periodicity phase (LPP)^94^ (Figure 1f). In the SPP, the lipid hydrocarbon chains can form three packing forms with the cross-section of the hydrocarbon alkyl chains indicating an orthorhombic, hexagonal, or liquid state (liquid crystal) arrangement (Figure 1e). The hexagonal and liquid crystal forms are characterized by greater mobility of the hydrocarbon chains as they are loosely packed and therefore have greater mobility and permeability compared to lipid chains in a orthorhombic arrangement.^95,96^ The thickness of the SPP depends on the amount of water contained in the SC, as with increasing hydration, up to 60% w/w, the length of the SPP increases linearly indicating the formation of a new aqueous phase between the SC lamellar lipid layers (directly affecting the increase in SPP phase length).^94^ Phase separation in the lamellar lipid region can lead to a rearrangement of the hydrocarbon lipid chains into micelles.

The study by Ogawa et al.^97^ cited previous findings^98^ and confirmed that a hydrated SC results in increased permeability to hydrophilic APIs. A study by Yamamoto et al.^99^ discussed the important role of the inhibition of transepidermal water loss through occlusion in the penetration of ketoprofen. Physiologically, the water content of the epidermis decreases from the viable epidermal layers of the *stratum spinosum* and *stratum granulosum* (about 70% by weight) to the SC. On average, the water content in the SC is 25%, but authors have reported different results which may have been influenced by measurement conditions as well as the individual nature of the epidermal barrier.^100^

Protein components account for 60–85% of the weight of SC. They are formed by keratin fibers, among which acidic type I keratin and neutral to basic type II keratin can be identified. Acidic keratins have a greater number of negatively charged amino acid side chains, such as aspartic or glutamic acid. Basic proteins have a greater number of positively charged side chains such as lysine, arginine, or histidine.^101^ It is known that under the influence of increasing humidity, the secondary structure of keratin changes from an α- to a β-helix conformation (Figure 1d). Keratin in α-helix conformation has a coiled-coil structure in which the side chains do not interact with water molecules.^102^ In the β-helix conformation, the side chains are exposed to the main chain of the protein so that water molecules have access to the peptide bonds and bind to them through hydrogen bonds.^102,103^

A study by Jokura et al. provided information that water molecules not bound to corneocyte proteins are part of the natural moisturizer factor (NMF).^104^ NMF further consists of amino acids, urea, lactic acid salts, pyroglutamic acid (PCA) and its salts, sugars, sodium, magnesium, potassium, calcium, chlorine, and phosphate ions.^105,106^ NMF form ionic bonds to keratin fibers which alter the elasticity of keratin by reducing intermolecular forces.^104^

The lipid mixture of the SC, organized in a double layer, provides protective properties to the epidermis and at the same time forms a barrier to API diffusion. It consists of ceramides (40–50% by weight), cholesterol (20–30%), cholesterol sulfate (2–5%), and free fatty acids (7–13%).^107^ Ceramides are particularly important in providing a barrier function to the epidermis. Ceramides in the SC are constructed from a sphingoid base, which can be sphingosine, dihydrosphingosine, or phytosphingosine and 6-hydroxysphingosine linked by an amide bond to the acyl residue of a fatty acid which can be nonsubstituted, α-hydroxylated, or ω-hydroxylated and contain an ω-linoleoyl group.^108−111^ Recently, 1-*O*-acylceramides have also been identified in the human SC, where their esterified fatty acid is attached to the headgroup of sphingosine in position 1. This component is described as one of the key components of the SC because its deficiency leads to impaired protective properties and increased water loss.^112^ It is generally accepted that ceramides are composed of a small polar head and two simple saturated hydrophobic aliphatic chains. The polar part (polar fragment, the head) consists of 2–4 hydroxyl groups and an amide group. This structure ensures the formation of a strong hydrogen bonding network that maintains the stability of the lamellar structure and the strength and barrier properties of SC.^113,114^

The free fatty acids in human SC generally consist of saturated (chain lengths ranging from C14:0 to C34:0) and unsaturated (C16:1 to C18:1, C30:1 to C36:1, C18:2) fatty acids.^115^ The most abundant group of fatty acids present in the SC (>50% of all SC fatty acids) are saturated, linear chain lipids with 16 to 30 carbon atoms with the most common being lignoceric acid (C24:0) and hexacosanoic acid (C26:0).^115−117^

The SC is not only a physical barrier, as its protective properties are enhanced by chemical and immunological factors. Scientific literature indicates that the presence of the skin microbiome, which forms the natural physiological flora and is responsible for both chemical and immunological factors.^118^ The skin’s physiological flora consists of microorganisms such as *Corynebacterium* species, *Cutibacterium acnes*, coagulase-negative *Staphylococcus* species including *Staphylococcus epidermidis*, and *Malassezia spp*. The important role of the skin microbiome in creating the skin’s protective barrier is supported by the fact that it promotes the differentiation and integrity of the epithelium. Additionally, some skin bacteria secrete sphingomyelinases, which are responsible for the production of ceramides in the SC.^119^ Furthermore, microorganisms that colonize the skin produce lipase enzymes that break down sebum triglycerides into free fatty acids. These free fatty acids strengthen the acidic nature of the skin, which limits the colonization of pathogenic microorganisms on the skin.^120^ The physiological pH of the skin surface is acidic, falling within the pH range of 4.1 to 5.8. However, as one moves closer to the living layers of the epidermis, the pH increases to neutral levels of pH 7 to 7.4. In a low pH environment, fatty acids exist in a nonionized form, which causes minimal repulsion of lipid head groups and promotes the formation of lamellar structures, ultimately affecting the integrity of the protective barrier. Increased pH of the skin surface is associated with impaired function of the protective barrier of the SC.^121^

To summarize, the SC forms a hydrophobic, nonpolar protective barrier, which is determined by the homeostatic composition and quantity of the individual components of the SC. Fluctuations in the quantity of SC components as well as increased hydration of this layer significantly alter its properties. Regarding the transdermal administration of APIs, the information above is a valuable clue for researchers, as it encourages the search for permeation-enhancing agents that can temporarily affect the chemical composition and hydration of the SC. By doing so, the permeability of the SC to APIs can be increased, facilitating their transdermal delivery.

## 3 Natural Components of the SC Are Compounds That Regulate the Permeability of APIs through the Skin

Substances applied externally that penetrate the structure of the SC interact with its components. Substances containing compounds that physiologically occur in the SC, such as ceramides, cholesterol and its sulfate, and fatty acids affect the proportions of their counterparts resulting in supplementation of their deficiencies.^122−128^ Conversely, they may disrupt the natural quantitative balance of lipid components to induce an increase in skin permeability. It has been found that changes in the composition and chain length of fatty acids present in their free forms as well as bound to SC ceramides change their melting point and membrane permeability.^129^ The potential to modulate the barrier properties of the SC appears to be helpful in assisting the transport of APIs across the skin.^130^

### 3.1 Sterols

In the SC, the ratio of cholesterol (Table 2, item 1) to cholesterol sulfate (Table 2, item 2) is important in maintaining the SC barrier, as it is indicated that with increasing amounts of cholesterol sulfate, an increased fluidity of the lipid fraction is observed leading to a greater permeability of the SC,^131,132^ while at the same time, the chains of fatty acid residues or chains of acyl groups and ceramides in the SC remain rigid.^131,132^ Cholesterol sulfate, by weakening the barrier functions, may serve as a substance that facilitates API permeation.^132^ The explanation for the observed effects was based on an analysis showing that the polar, acidic, sulfate group of cholesterol sulfate exhibits a stronger hydrogen bonding capacity compared to the rest of the nonpolar, hydrophobic components of the lipid matrix. Cholesterol sulfate groups, as a result of repulsive electrostatic forces and the solvation effect of charged atoms, increase the hydration region between lipids creating a pathway for hydrophilic APIs.^131,132^ Furthermore, cholesterol sulfate is a highly amphiphilic molecule with the ability to penetrate cell membranes by diffusion.^133^

**Table 2:**
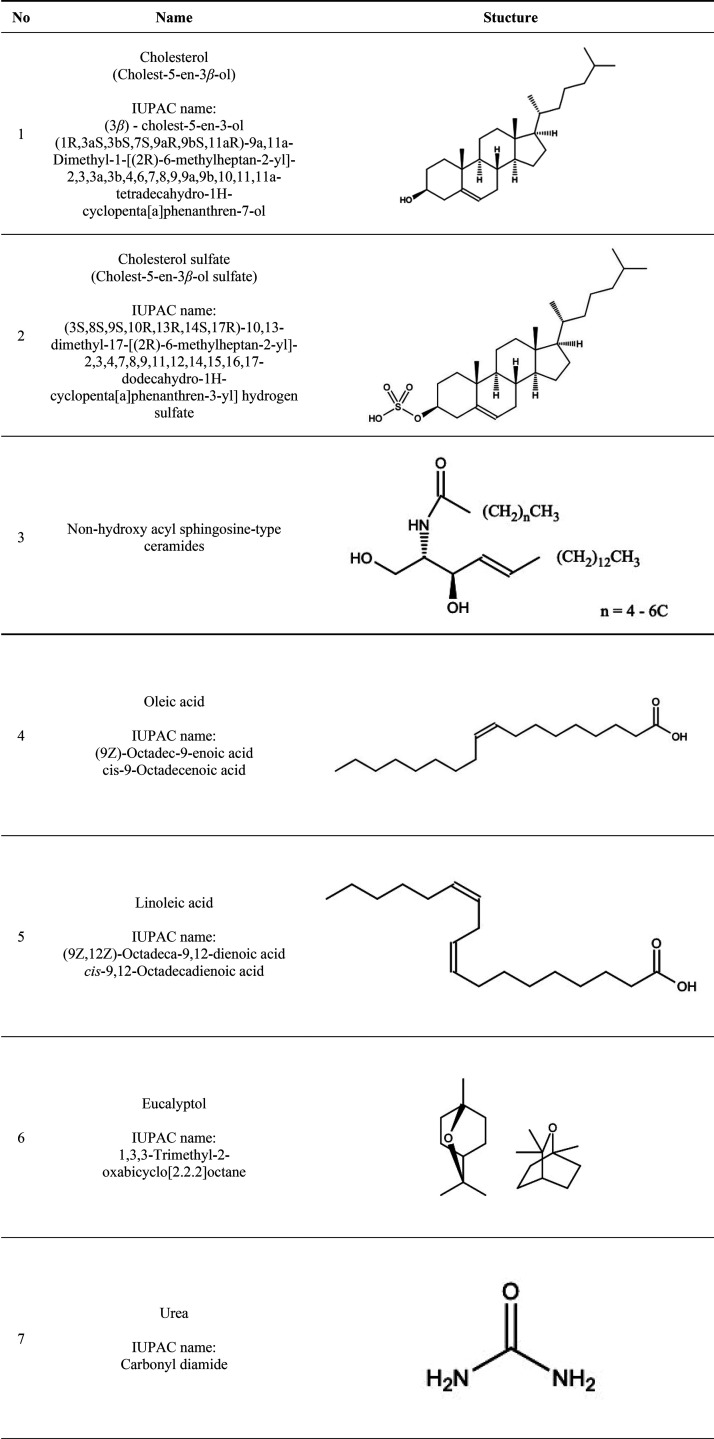

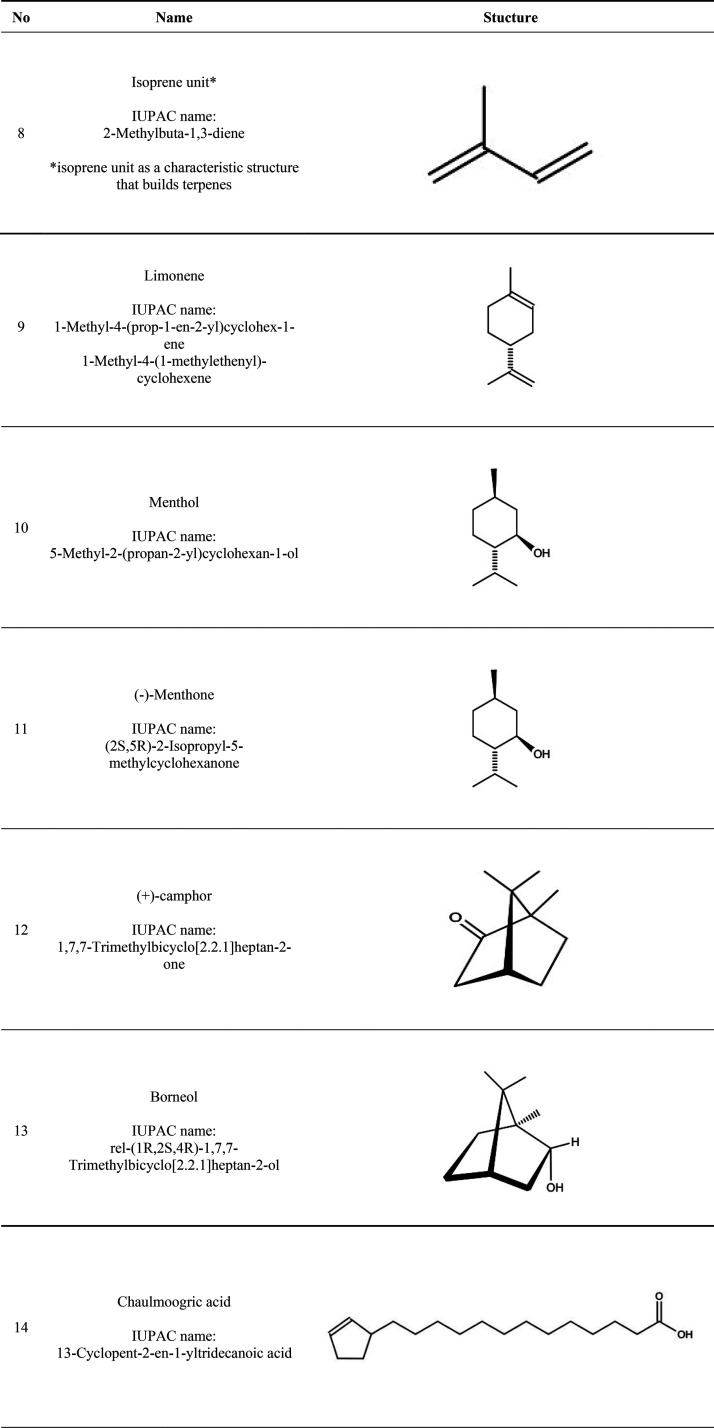
Structural Patterns of Natural SC Components Regulating Skin Permeability of APIs (Entries 1–5 and 7) and Natural Compounds with the Permeation-Enhancing Potential of APIs (Entries 6 and 8–16)

### 3.2 Ceramides

Current scientific research does not directly describe topically applied ceramides as penetration enhancers for APIs, but their role in modulating the barrier properties of the epidermis has been confirmed. Studies have evaluated the influence of the fatty chain and the size of the polar head of ceramides on the permeability of externally applied substances, which suggests the potential for ceramides as future penetration enhancers for APIs.^113,134−136^ In healthy human SC, ceramides contain fatty acid residue chains of more than 22 carbon atoms (Table 2, item 3).^108,137,138^ Shorter ceramide chains are observed in dermatoses that damage the skin barrier, such as atopic dermatitis, lamellar ichthyosis, Netherton syndrome, psoriasis, and autosomal recessive congenital ichthyosis.^113,134−136^ The effect of ceramide acyl chain length on SC barrier integrity has been confirmed in studies,^139−141^ in which it was observed that replacing the acyl chain in nonhydroxy acyl sphingosine-type ceramides (CER NS) of C24 length with a shorter one (C4–6) resulted in a significantly increased SC lipid permeability. An *in vitro* permeability study by Školova et al.^56^ using Franz diffusion cells of 5% theophylline (180 g/mol, LogP = 0) and 2% indomethacin (358 g/mol, LogP = 4.3) showed that replacement of CER NS (C24) with CER NS (C6) resulted in an increase of approximately 3.5 times the permeation of theophylline and nearly 6.5 times that of indomethacin. The paper by Uche et al.^57^ evaluated the effect of substituting a CER NS with a 16-carbon chain at the expense of CER NS (C24) on lipid membrane permeability of ethyl-*p-*aminobenzoate (E-PABA). An *in vitro* study using PermeGear in-line diffusion cells showed that the amount of API permeated through the lipid membrane increased with CER NS (C16) concentrations to 3.5 times more API at 50% CER NS (C16) concentrations and 6 times more API at 75% CER NS (C16) concentrations compared to long-chain ceramide membranes. The increased permeability of the model lipid membrane consisting of ceramides, fatty acids, cholesterol, and cholesterol sulfate was accompanied by an increased distance between the LPP and a change in the spatial arrangement of the lipids, as some of the lipid chains formed a hexagonal phase at the expense of the rhomboid phase as confirmed by SAXS (small-angle X-ray scattering). The increased space between the SC lipid layers and decreased packing density of the lipid chains explain the easier penetration of APIs. Table 1 (items 1 and 2) presents the permeation effectiveness of APIs used with ceramides in TTDs.

### 3.3 Fatty Acids

#### 3.3.1 Oleic Acid

The most frequently used fatty acid in TDD systems is the unsaturated oleic acid (OA), *cis*-octadecenoic acid (Table 2, item 4). An *ex vivo* study on rat skin using Raman spectroscopy provided information on the disorganizing effect of OA on SC lipid conformations.^53^ The double bond in the configuration cis of the OA structure, disrupts the organization of the alkyl chains of the lamellar lipids leading to separation and increased fluidization of the lipid bilayers.^142,143^*In vitro* study using in-line Bronaugh flow-through diffusion cells showed that the flow of the protein interferon alpha-2B drug (19 kDa) through the SC was not enhanced in the presence of a solution containing 10% OA in propylene glycol. However, a separate liquid phase in the SPP region of the lamellar lipids was simultaneously observed in an *ex vivo* study on human skin using small- and wide-angle X-ray scattering analysis, which formed a potential pathway predisposing to the penetration of API molecules.^58^ Research into the mechanism of action of OA dates back to the 1990s, when Naik’s research team used attenuated total reflectance infrared spectroscopy. It was shown that a 5% solution of OA in ethanol applied to the human skin *in vivo* creates a separate liquid phase leading to the separation of the lamellar lipid bilayers and causes fluidization. This finding indicated that an observed effect of changing the conformation of epidermal lipids predisposes it to the permeation of small molecules.^144^ Similar conclusions were presented by Jiang et al. following an *in vivo* study on rat skin in which 10% OA in propylene glycol was applied.^145^

An *in vitro* permeation study using Franz-type diffusion cells with a lipid membrane mimicking the quantitative and qualitative composition of human SC in the presence of 10% OA showed that, of the three model drugs used, 10% urea (60.06 g/mol, LogP = −2.11), 2% caffeine (194.19 g/mol, LogP = −0.07), and 0.1% diclofenac sodium (318.14 g/mol, LogP = 4.28), urea was the most hydrophilic substance and had the fastest diffusion rate. However, there were no differences in lipid membrane permeability in the presence and absence of OA, indicating the natural penetrating properties of urea.^59^ Therefore, it can be inferred that OA does not provide significant API permeation-enhancing properties, despite its disorganizing effect on lipid fractions. The *in vitro* permeation effect on Franz diffusion cells using a rat skin-insulin model was also assessed with three different fatty acids: OA (C18:1, LogP: 7.64), linoleic acid (C18:2, LogP = 7.05), and linolenic acid (C18:3, LogP = 6.46). OA was shown to have the best permeation enhancement profile of these three fatty acids, but not enough to be considered an insulin permeation facilitator. Linolenic acid (Table 2, item 5) reduced the permeation of insulin through the skin,^60^ which correlates with reports by other authors on the lipid-barrier-rebuilding properties of this fatty acid.^51,146^ The paper explains that this phenomenon is due to the lower oil/water partition coefficient, higher structural rigidity related to the number of double bonds, and higher surface tension of linolenic acid compared to acids with fewer double bonds.^60^

Currently, OA has also started to be used as a component of carrier formulations, e.g., in micro- or nanoemulsions, whose dermal absorption-enhancing properties for APIs are used in various medical areas.^147−151^

The work of Abd et al.^77^ evaluated an *in vitro* Franz diffusion cell model mimicking human skin to determine the permeation efficiency of 2% minoxidil (LogP = 1.24) incorporated into an oil-in-water (o/w) nanoemulsion with OA or eucalyptol (Table 2, item 6) entering the oil phase. Both nanoemulsion formulations (the one with OA and the one with terpene) were shown to increase the amount of API penetration through the SC and its solubility compared to the API alone, with a greater efficacy observed in the formulation containing eucalyptol. Nanoemulsions with OA and eucalyptol were also found to be effective in increasing the permeation of 3% caffeine and 2% naproxen.^78^

A nanoemulgel with OA was used to deliver caffeic acid from propolis (*Apis trigona*). An *in vitro* permeation study on Franz diffusion cells through rat skin showed that the highest API retention was obtained using OA at a concentration of 2.5%, compared to 5% and 1.25%.^79^ Lai et al.^80^ published results showing a 4-fold more effective (compared to a control trial without carriers) delivery of 2,3,5,4′-tetrahydroxystilbene 2-O-β-d-glucoside (extracted from *Polygonum multiflorum*) using OA-containing vesicles loaded into a complex gel. The permeation-enhancing properties of OA were modified by the use of carrier and gel forms in TDD systems. Table 1 (items 3–8 and 35–39) presents the permeation effectiveness of APIs used with oleic acid in TTDs.

### 3.4 Urea

Urea, also known as carbamide, is an acyclic, polar compound containing a carbonyl group attached to two amine groups (Table 2, item 7). It is naturally included in human NMF (7% of NMF composition).^152,153^ It is a compound that exhibits hygroscopic and keratolytic properties in a concentration-dependent manner. In formulations for external application, a 2–10% concentration shows a moisturizing effect, a 10–20% concentration shows a moisturizing, keratolytic, and API-permeation-enhancing effect, and a 30–50% concentration no longer shows a moisturizing effect.^153,154^

An *in vitro* human epidermal barrier permeation study on Franz diffusion cells under infinite dosing conditions showed that urea had a higher permeation rate than more hydrophilic substances like glycerol and mannitol but less than the more lipophilic estradiol. In comparison, under finite dosing conditions, the amount of urea permeated was the highest among substances used in the study, which according to the authors was related to the difference in the skin hydration status between the two experiments, i.e., fully hydrated skin in the infinite dosing technique and partially hydrated skin in the finite dosing technique, the type of solvent, and the interactions between it and the substance and membrane used for the study.^155^ The differences between *in vitro* testing under infinite dosing and finite dosing conditions were described by Franz,^156^ which indicated that greater reliability of *in vitro* test results is obtained with the finite dosing technique. Intarakumhaeng et al. found that nonvolatile substances with a molecular weight up to 60 g/mol, as in the case of urea, show a relatively high percentage of a penetrating dose.^155^

The mechanism of action responsible for the potential permeation enhancement of SC proposed by Mueller et al. is based on its ability to bind water and import it into the corneocytes. A large amount of bonded water can lead to an increase in cell volume of up to 50% and an increase in osmotic pressure.^157^ Water can also accumulate between corneocytes in the SC, which can affect the barrier function of the skin. In the lipid bilayer region, water can also disrupt the local electrostatic interactions and lead to the formation of pores in the lipid membrane. This can occur due to the reorientation of hydrophilic lipid headgroups toward the center of the bilayer and the formation of inverted micelles.^158,159^

Additionally, varying the water content of the SC can potentially alter the degree of ionization of fatty acids, which are commonly assumed to exist in a nonionized form.^159^ NMF results published by Pham et al. confirmed that the final segments of the keratin structure, on which glycine and serine residues are located, are fluidized in the presence of urea.^160^ Urea is identified in the literature as a chemical “denaturant” that alters the structure of proteins indirectly by affecting the hydrogen bond network of bound water at the boundary of the protein.^161−163^ Another concept concerning the denaturing effect of urea has also been proposed, stating that urea directly interacts with the protein by breaking intramolecular hydrogen bonds.^164^

In an *in vitro* study, a gel with 10% urea increased transdermal caffeine delivery by almost 50%, at a level similar to the permeation-enhancing effect of the surfactant sodium laureth sulfate. Furthermore, the gel formulation appeared to be more effective in promoting caffeine absorption than the use of an emulsion.^61^ Shams et al.^81^ used urea at a concentration of 2 M (12.1%) together with the cyclodextrin derivative hydroxy propyl-β cyclodextrin (HP-β-CD) to deliver recombinant human growth hormone (rhGH) to the deeper layers of the skin. The use of two permeation enhancers showing a synergistic effect had a positive effect on the delivery of the API, with the greatest amount of substance permeated after 120 min. The synergistic effect was due to the protective effect of HP-β-CD on the structure of rhGH in the presence of urea, such that without HP-β-CD the activity of the hormone decreased. Higher concentrations (4–8 M) of urea resulted in significantly lower levels of rhGH in the skin, which, according to the authors, could be due to the denaturing effect of urea. Table 1 (item 9) presents the permeation effectiveness of APIs used with urea in TTDs.

## 4 Natural Compounds with the Penetration-Enhancing Potential of the API

### 4.1 Terpenes

Terpenes represent a group of compounds with the general formula, (C_5_H_8_)_*n*_, consisting of two or more five-carbon isoprene units (IUs) (Table 2, item 8). They are classified according to the number of isoprene units in the carbon skeleton: monoterpenes (2 IUs), sesquiterpenes (3 IUs), diterpenes (4 IUs), sesterterpenes (5 IUs), triterpenes (6 IUs), tetraterpenes (8 IUs), and polyterpenes (>8 IUs). The structure of terpenes can be formed either by carbon chains or carbon rings. These arranged carbon rings can be further classified as monocyclic (one carbon ring) and successively bicyclic, tricyclic, etc. Acyclic compounds do not contain a carbon ring.^165^ Isoprene is one of the most abundant volatile hydrocarbons produced by living organisms in the world including bacteria.^165,166^

Terpenes have a high LogP which is indicative of their lipophilic properties determining their solubility in SC lipids. However, the presence of polar and nonpolar groups in the terpene molecule makes them exhibit the potential to promote the permeation of both hydrophilic and lipophilic APIs.^167,168^ The different degrees of lipophilicity of terpenes (e.g., LogP = 2.13 for camphor and LogP = 5.32 for nerolidol), structure (linear or ring), and presence of additional functional groups give them different permeation enhancement efficiencies.^169^

In section 3.3.1, we described eucalyptol because its use was related to the OA study.

An *in vitro* study showed a higher amount of permeation of the API haloperidol (375.9 Da, LogP = 3.36) across the human epidermal membrane in the presence of limonene (Table 2, item 9) (LogP = 4.45) than in the presence of the oxygen-containing terpenes linalool (LogP = 3.28) and 1,4-cineole (cineole LogP = 2.31 1,8-cineole LogP = 2.82). The work does not indicate exactly which terpene was used, which was explained by the facilitation of API solubility in the skin by limonene.^62^ FTIR (Fourier-transform infrared spectroscopy) results confirmed that limonene interacts with the alkyl chain region of SC lipids causing an increase in fluidization, as it relaxes the lamellar organization of the lipids.^170^

Zhu et al.^171^ evaluated seven oxygen-containing terpenes (1,8-cineole, citral, geraniol, linalool, menthol, terpinen-4-ol, and α-terpineol) at a concentration of 5%; both cyclic and linear forms were evaluated using the method of measuring skin electrical resistance (SER), the changes of which reflect the degree of skin integrity after application of compounds considered to be API permeation enhancers.^172,173^ Cyclic terpenes (1,8-cineole, terpinen-4-ol, menthol, and α-terpineol) were shown to reduce SER values and impair the skin’s barrier function to a greater extent than linear terpenes (linalool, geraniol, and citral). Molecular computer simulations indicated that the SER values for cyclic terpenes were associated with the formation of stronger hydrogen bonds with the polar head of SC ceramides^171^ creating a pathway for hydrophilic API penetration (LogP > 3), as well as a pathway for water to escape through the epidermis.^171,174^

In an *in vitro* study conducted in Franz diffusion cells, differences in the enhancement of ligustrazine hydrochloride permeation using the monocyclic monoterpenes menthol (Table 2, item 10) and menthone (Table 2, item 11) were assessed. Menthol contains a hydroxyl group attached to its ring and was found to be more effective than menthone with a carbonyl group. The hydroxyl group more easily forms hydrogen bonds with the amide group of ceramides and, as confirmed by FTIR analysis, menthol exhibits stronger epidermal lipid-extracting activity.^63^ In the study of Huang et al.,^175^ the simulation of skin permeability using molecular dynamics confirmed the interaction of menthol with ceramide 2 by forming hydrogen bonds in a single-component bilayer model, which facilitated the penetration of quercetin. The study showed that quercetin tended to localize in the area of the polar heads of the lipid bilayer, which created barriers for its deeper penetration through the hydrophobic region of the lipids. By hydrogen bonding with menthol, quercetin reduced its chances of interacting with ceramide. Meanwhile, menthol inserted itself into the lipid bilayer, breaking the hydrogen bonds between ceramides and facilitating the diffusion of quercetin. However, the effect of the interaction of menthol with quercetin on the SC should be tested on a more complicated bilayer model that reflects the natural composition of the SC lipid mixture.

An *in vitro* rat skin transdermal permeation study using a TK-20B diffusion apparatus showed that (+)-camphor (Table 2, item 12) (monoterpene, LogP = 2.13) at a concentration of 3% increased the permeation of APIs with different lipophilicities: indomethacin (LogP = 3. 80), lidocaine (LogP = 2.56), aspirin (LogP = 1.23), antipyrine (LogP = 0.23), tegafur (LogP = −0.48), and 5-fluorouracil (LogP = −0.95). At the same time, camphor was found to increase the permeation efficiency of the APIs linearly with decreasing LogP values, as the highest amount of API permeation through the skin was observed for hydrophilic APIs (LogP of approximately 0). Camphor was found to increase the partitioning of the API in the SC, i.e., to increase the release of the API from the carrier into the SC.^176^ This is the first step in drug delivery through the skin, resulting in a concentration gradient as molecules diffuse into deeper layers of the epidermis and dermis.^177^ Camphor also extracts some of the lipids and disrupts the molecular organization of SC lipids, as confirmed by FTIR analysis.^64^ Borneol (Table 2, item 13), a monoterpene with similar lipophilicity (LogP = 2.71) and structural similarity to camphor, was reported by Dai et al.^65^ Franz diffusion tests showed that at a concentration of 0.54%, borneol inhibited the penetration of the lipophilic API osthole (LogP = 3.8), while increasing API penetration was observed at the higher borneol concentration of 1.02%. TEM (transmission electron microscope) imaging provided information on the disorganizing effect of 0.54% borneol on lipids in rat skin, while a concentration of 1.02% resulted in the complete destruction of the lamellar lipid arrangement. Using CGMD (coarse-grained molecular dynamics), it was observed that borneol at concentrations above 10–15% disorganized the arrangement of lipid alkyl chains and caused the extraction of some lipids and formed aqueous spaces and inverted micelles, while concentrations up to 10% localized to the space of lipid alkyl chains without affecting their structure. The disorganizing and extracting effects on SC borneol lipids at concentrations of 1%, 3%, and 5% were confirmed using ATR-FTIR (attenuated total reflectance–Fourier-transform infrared spectroscopy) by Yi et al.^178^ Thus, the disorganizing effect on the lamellar lipid structures is not sufficient for the penetration of lipophilic compounds when using borneol at concentrations below 1%.

The study by Kopečná et al.^66^ describes a novel class of penetration enhancers, which are a combination of an amino acid derivative with various mono- and sesquiterpene alcohols (namely, 6-(dimethylamino)hexanoic acid with citronellol, geraniol, nerol, farnesol, linalool, menthol, borneol, and carveol esters). The researchers tested the effectiveness of these enhancers in delivering two different APIs, theophylline (MW = 180 g/mol, LogP = −0.02) and hydrocortisone (MW = 362 g/mol; LogP = 1.61), through human skin *in vitro*. Among all the terpene alcohols used, citronellyl 6-(dimethylamino)hexanoate was found to be the most effective at a concentration of 30 mM. Importantly, these enhancers did not show any cellular toxicity *in vitro*, and their mechanism of action was found to be based on the fluidization of epidermal lipids, as confirmed by FTIR analysis on the isolated human epidermis.

Terpenes are the main components of essential oils. Natural essential oils are extracted from the herb, leaves, flowers, and fruit. The methods used to extract essential oils from the plants include supercritical fluid extraction, subcritical liquid extraction, solvent-free microwave extraction, hydrodistillation, steam-distillation, hydrodiffusion, and solvent extraction. Essential oils include nitrogen- and sulfur-containing compounds (isocyanates, e.g., allyl isothiocyanate), aromatic compounds (benzene derivatives, e.g., eugenol), and others, including long-chain unbranched compounds.^179,180^ In an **in vitro** study on rat skin, the permeation of naproxen sodium from a gel matrix in the presence of an absorption promoter in the form of 0.5% essential oil of *Lavandula angustifolia*. The use of this oil formulation allowed the API to be delivered to the skin at a higher concentration (222.19 ± 24.87 μg/cm^2^) compared to naproxen gel alone (107.65 ± 6.38 μg/cm^2^). The main constituents of lavender oil, as confirmed by gas chromatography–mass spectrometry (GC-MS) analysis included 1,8-cineole (22.3%), linalool (11.2%), camphor (7.9%), β-pinene (5.8%), α-terpineol (4.9%), α-pinene (4.6%), terpinen-4-ol (4.2%), and borneol (4.0%).^67^ An *in vitro* rat skin permeation study showed that essential oils at a concentration of 3% extracted from *Radix Angelicae sinensis* [*Angelica sinensis* (Oliv.) Diels (*Umbelliferae*)], *Rhizoma chuanxiong* [*Ligusticum chuanxiong* Hort. (*Umbelliferae*)], *Rhizoma cyperi* [*Cyperus rotundus* L. (Sedge)], *Cinnamomum cassia* Presl. (*Lauraceae*), and *Flos caryophylli* [*Eugenia caryophyllata Thunb.* (*Myrtaceae*)] are effective when used alone in facilitating the penetration of ibuprofen. The main constituents of these oils included ligustilide (79.32%), ligustilide (41.00%), (*E*)-cinnamaldehyde (83.30%), and eugenol (80.22%), respectively.^68^ In contrast, the essential oil of *Sinapis alba* L. when used at concentrations of 0.5%, 2%, and 5% (with the highest efficacy at 5%) showed promising permeation enhancement properties for the following APIs with different lipophilicities: 5-fluorouracil (LogP = −0.95), paeonol (LogP = 2.054), and osthol (LogP = 3.85). The main constituents of the oil were 3-butenenitrile (16.62%), allyl isothiocyanate (57.02%), and isothiocyanato cyclopropane (17.46%).^69^

The compounds that make up the majority of essential oil compositions do not necessarily interact with the SC constituents and are responsible for the permeation-enhancing effects of APIs, as confirmed by Nowak et al.^70^ The essential oil from *Epilobium angustifolium* L. consists mainly of cosanes (23.70%), 5-methyldocosane (14.95%), caryophyllenes (9.22%), and oxygen derivatives of caryophyllenes (11.30%). In an *in vitro* study on Franz cells, the oil promoted the penetration of ibuprofen, lidocaine, and caffeine through pig skin. Analysis of the composition of the pig skin and acceptor fluid after 24 h by GC-MS analysis revealed the presence of α-terpineol, (*S*)-carvone, thymol, anethole, secalciferol, and trimethylpentadecan-2-one in the skin, which accounted for 1.94–4.54% of the oil composition, while no compounds were found in the acceptor fluid.^70^ The results suggest that the compounds present in the skin are responsible for the beneficial effects of API penetration.

Due to the difficulty in identifying which components are responsible for the penetration-enhancing effect, it may be considered fair to compare the effect of a śś of essential oil with individual compounds isolated from the oil.^169^ A question that is worth considering is whether a mixture of these compounds shows a synergistic effect resulting in improved API absorption through the skin. Oil extracted from *Ledum palustre* L. var. *angustum* N. Busch was used in an *in vitro* horizontal diffusion cells study to evaluate its efficacy in facilitating the permeation of donepezil hydrochloride. The compound facilitating the penetration of the API was cuminaldehyde, which accounted for 5.72% of the oil composition. Cuminaldehyde, used alone in the same study, was found to be 2 times more effective than the oil alone. Terpenes included in the oil were sabinene (33.40%), 4-terpineol (20.33%), and *p*-cymene (18.31%), but when used alone, they showed little or no efficacy.^71^

Table 1 (items 10–29) presents the permeation effectiveness of APIs used with terpens and essentials oils in TTDs.

### 4.2 Fatty Acids

Plant fats such as tri-, mono-, and diglycerides, free fatty acids, phosphatides, sterols, and fatty alcohols occur in nature in plant tissue.^181^ They are most abundant in seeds, pulp, stone fruit, tubers, and sprouts. The main source of plant lipids are oilseed-producing plants such as sunflower, soybean, and rapeseed, oilseed-producing fruits such as olive, coconut, and palm, oilseed tubers such as peanuts, or oilseed germ such as maize. Methods for extracting the lipid compounds are based on chemical extraction, supercritical fluid extraction, steam-distillation, mechanical extraction, and most commonly mechanical pressing by which vegetable oil is extracted.^181,182^ Volatile essential oils and fatty vegetable oils differ in the content of the compounds predominantly present in the same plant source such as terpenes as well as other bioactive compounds, such as flavonoids.^182^ Plant oils have been found to influence the penetration of active substances through the skin, and fatty acids are mainly responsible for this effect.^72^

Results published by Cizinauskas et al.^72^ showed that of all the oils used (0.5% w/w) olive oil, soybean, coconut, avocado, sea-buckthorn pulp, and raspberry seed oils contained the same fatty acids in different proportions: C16:0 (palmitic), C18:0 (stearic), C18:1 (oleic), C18:2 (linoleic), and C16:1 (palmitoleic). Of these, only soybean oil and olive oil increased dihydroquercetin penetration (LogP < 3) *in vitro* using Bronaugh-type flow-through diffusion cells. The API in the presence of soybean oil was localized in the epidermis and dermis, while with olive oil the API penetrated deeper by localizing only in the dermis, as confirmed by TOF-SIMS (time-of-flight secondary ion mass spectrometry) analysis *in vivo* using human skin. The unsaturated fatty acids from the oils used in the study penetrated into the deeper layers of the epidermis and dermis, but at the same time, no correlation was observed in the study between the concentration of individual fatty acids and the effect of increased API penetration through the skin. According to the authors, this may be due to the presence of other components in the oil, not detected by the GC-MS analytical method used (after derivatization into methyl esters), and the synergistic effect of a mixture of penetration enhancers. Using flurbiprofen, which is more lipophilic than dihydroquercetin (LogP = 4.16), olive oil proved to be the best penetration enhancer compared to avocado oil, coconut oil, and oils of animal origin: emu and crocodile. This effect correlated with the highest amount of oleic acid (OA) in the oil formulation (76%). The second highest amount of OA was avocado oil (68%). However, it was coconut oil that showed greater penetration enhancement effects despite containing a high concentration (52%) of saturated, short-chain lauric acid (C12:0).^73^ An *ex vivo* study by Singh et al. using porcine skin and a Keshary–Chien glass diffusion target showed that seed kernel oil of *Mesua ferrea* Linn. at a concentration of 15% significantly increased the penetrating amount of diltiazem hydrochloride. FTIR spectra showed changes in the peak positions of the methylene groups of SC lipids and the amide groups in SC keratin indicating disordered lipid organization, lipid fluidization, and an altered conformation of keratin fibers in mesua oil-treated skin samples. Scanning electron microscope (SEM) imaging of the epidermis confirmed the disruption of protein structure in the SC.^74^ The mechanism of action was not correlated with the composition of the oil; however, other researchers have provided information on the content of OA, stearic acid, linoleic acid, palmitic acid, myristic acid, and arachidic acid,^183^ as well as coumarins, terpenoids, phenolics, and flavonoids in the oil.^184^

Another *in vitro* Franz diffusion cell study showed that oil extracted from *Punica granatum* seeds at concentrations of 2.5%, 5.0%, and 10% increased the amount of trans-resveratrol penetrating pig skin by 1.25, 2.25, and 3.14 times, respectively, compared to a control sample without oil. Analysis of the oil composition by GC-MS showed a composition of punicic acid (C18:3, 73.93%) with OA (C18:1), eicosenoic acid (C20:1), and linoleic acid (C18:2) which together accounted for 13.46% of the oil composition.^75^ A study^82^ demonstrated the penetration-enhancing potential of chaulmoogra oil extracted from the seeds of a tree from the genus *Hydnocarpus* and family *Flacourtiaceae.*(185) A nanoemulsion containing chaulmoogra oil at approximately 84 μg/mL, Tween surfactant at approximately 90 μg/mL, and cosurfactant at approximately 2 μg/mL was developed for the transdermal delivery of methotrexate in the dermatological treatment of psoriasis. Drug retention studies showed that higher amounts of the API were retained in the epidermis and dermis, the layers that are mainly affected by psoriasis. Fluorescent microscopy analysis of skin cells also confirmed the presence of the drug in the deeper layers of the skin. In an *in vivo* clinical evaluation of the efficacy of this psoriasis treatment using the Psoriatic Area Severity Index (PASI) score, the nanoemulsion reduced the PASI by approximately 95% after 28 days. Chaulmoogra oil alone reduced the PASI by an average of 46%.^82^ Chaulmoogra oil is characterized by the presence of predominantly cyclopentenyl fatty acids: chaulmoogric acid (C18:1) (Table 2, item 14), hydnocarpic acid (C16:1), and gorlic acid (C18:2) (Table 2, item 15) containing a five-carbon ring with one unsaturated bond attached to the carbon chain.^186^ These fatty acids exhibit unusual biological activity against acid-resistant bacteria, and the oil itself was once used as a API to treat leprosy in humans.^187,188^ Cyclopentenyl acids from chaulmoogra oil have been shown to incorporate into triacylglycerols and cell membrane phospholipids to further disrupt membrane processes leading to the inhibition of *Mycobacterium vaccae* proliferation.^185,189^

Table 1 (items 30 to 33) presents the permeation effectiveness of APIs used with oils in TTDs.

### 4.3 Polysaccharides

Polysaccharides are naturally occurring polymers that are found in plants (e.g., starch, cellulose, and pectin), marine sources (e.g., agarose, alginate, chitosan, and carrageenan), microorganisms (e.g., dextran and pullulan), and animals (e.g., hyaluronic acid (HA), chondroitin sulfate, and heparin). Some of them have been described in the literature as compounds showing the potential to modify the skin barrier which supports their use in TDD, mainly for hydrophilic active ingredients.^190,191^ They have beneficial properties that make them widely used in the pharmaceutical industry. They exhibit susceptibility to chemical modification, sensitivity to environmental changes, and ability to swell in aqueous environments, and they are nontoxic, readily available, and biodegradable. These natural polymers via chemical or physical cross-linking form hydrogels, which are used in TDD. Most commonly, polysaccharides are physically cross-linked by means of electrostatic interactions, hydrophobic interactions, and ionic cross-linking supported by multivalent ions, van der Waals forces, or host–guest complexes.^192^

Mucilage is a complex heteropolysaccharide, which in contact with water becomes a viscous gel with a slimy appearance. It is extracted from plant parts such as fruits, pods, seeds, flowers, and leaves. The mucilage structure consists of hydrophilic groups namely −OH, −CONH–, −CONH_2_, and −SO_3_H entities with the ability to form noncovalent bonds with biological tissue.^193^ Saidin et al. extracted mucilage from fresh leaves of *Hibiscus rosa-sinensis* L. When applied to rat skin at concentrations of 1%, 1.5%, and 2%, it resulted in extraction and fluidization of corneocyte lipids and proteins and a conformational change in keratin SC proteins, as confirmed by ATR-FTIR analysis. Moreover, spectra analysis of the peak characteristic of the OH and NH groups showed the formation of hydrogen bonds between the gel components and SC ceramides, indicating a mechanism of action similar to terpenes (formation of new polar pathways for API diffusion). An *in vitro* permeation study using vertical diffusion cells showed an enhanced diffusion of caffeine in the presence of *Hibiscus* leaf mucilage with the best effect occurring at a concentration of 2%. The SEM image of the morphology of skin treated with *Hibiscus* gel showed an increase in skin smoothness after the application of the 2% gel compared to skin treated with a caffeine solution alone. The smooth surface of the SC permeation area had a reduced diffusional resistance to API transport.^76^ The natural saccharide gel also exhibited controlled drug-release properties. Mucilage extracted from the fruit of *Ficus reticulata* L. acted as a matrix in transdermal patches with properties that delayed the release of the API diltiazem hydrochloride into the SC.^83^ Mucilage extracted from *Ficus carica* L. also acted as a matrix in transdermal patches delaying the release of diclofenac sodium.^84^ Mucilage from *Colocasia esculenta* (taro) corms was combined with hydroxypropyl methylcellulose, which together served as a matrix in transdermal patches. An *in vitro* study demonstrated that these patches provided a safe, nonirritating control system for the release of diltiazem hydrochloride through the skin. Drug release slowed over time as the concentration of mucilage in the formulation increased.^85^

Hyaluronic acid (HA) is a linear glycosaminoglycan consisting of *N*-acetyl-d-glucosamine and d-glucuronic acid (Table 2, item 16) that is naturally found in the extracellular matrix of human connective tissue.^194^ For pharmaceutical and cosmetic purposes, it is extracted by microbial fermentation of microbial sources such as *Streptococcus zooepidemicus* and *Corynebacterium glutamicum.*(195) HA is used in a variety of drug delivery systems including nanoemulsion hydrogels, microemulsions, nanostructured carriers, and microneedles.^196,197^

Hydrogels form a three-dimensional network with a porous morphology. Due to the swelling and water-attracting properties of natural polymers, the hydrogel pores form a reservoir for water molecules or drug solutions. Drug release occurs under the influence of environmental changes such as pH, temperature, the presence of enzymes, and reactive oxygen species.^198,199^ HA is frequently used in pH-sensitive transdermal systems, such as hydrogels, as the presence of a COOH carboxyl group that dissociates at a pH equal to 6.7 creates a negatively charged COO– group that contributes to the release of the API into the skin through electrostatic interactions.^87^ The potential of HA-based hydrogels has been used in TTD systems for polar phenolic compounds. A study by Kong et al. indicated that hydrogels using HA (MW = 800 kDa) and another natural plant polymer, hydroxyethyl cellulose, facilitated the *in vitro* permeation through rat skin of the phenolic compound isoliquiritigenin extracted from *Glycyrrhiza uralensis.*(86) In a study by Kim, a hydrogel-based on HA (MW = 0.48 MDa) and poly(*N*-isopropylacrylamide) was found to be effective in transporting luteolin into the epidermis and dermis, as confirmed by UV–vis spectrophotometric analysis of *in vitro* drug-penetrated skin samples.^87^ The transport of proteins through the skin is hindered mainly due to their hydrophilicity and high molecular weights.^200^ The potential to enhance the permeation of the protein drug bovine serum albumin (66 kDa) hydrogel with 5% HA with different molecular weights of 5 kDa, 100 kDa and 1 MDa were evaluated.^88^ Using fluorescence resonance energy transfer (FRET)-FLIM, it was observed that low-molecular-weight HA (5 kDa) cotransported with the protein drug into viable epidermal layers, i.e., *stratum basale* and *stratum spinosum*, which was not observed with the higher HA molecular weights of 100 kDa and 1 MDa.^88^ Furthermore, the relationship between HA molecular weight with the ability to interact with SC proteins was confirmed by FTIR. Low-molecular-weight HA dramatically increased skin hydration and caused a conformational change in keratin structure from an α-helix to a β-sheet, which affects the organization of the lipid bilayer in the SC and the permeability to API. This effect was not observed for 100 kDa HA, which only increased the hydration of the SC.^88^ The SC penetration of HA is limited by its molecular weight, which can range from 5.000 to 5.000 000 Da. Raman microimaging of human skin sections provided information that only low-molecular-weight HA (20–300 kDa) was able to cross the SC barrier. In contrast, the SC barrier is impermeable to high-molecular-weight HA of 1.000–1.400 kDa.^201^ A new promising solution to improve the penetration of HA through the skin was proposed by Yan et al. The solution involves combining HA (in this study, HA with a molecular weight of 50 kDa) with a binding peptide called HaPP and a peptide called Pep-1. Together, these peptides promote the penetration of HA into the dermis.^202^ These penetrating peptides linked to HA are covered by patent application No. CN107226846B. In the discussed study by Yan et al., it has been suggested that the discussed solution opens the possibility of potentially combining HA with APIs.

Kawar et al. published information on a new type of liposome with a gel core formed from HA (hyaluosomes). Hyaluosomes were three times more effective in delivering ketoprofen through porcine skin *in vitro* compared to conventional liposomes (60 μg/h versus 20 μg/h).^89^ The beneficial effects of HA in enhancing API permeation are also exploited in the modification of other liposomal carriers and ethosomes to form phospholipid-ethanol complexes with HA.^203^ Fluorescence microscopic imaging of rat skin showed that ethosomes containing sodium forms of HA (MW = 150 kDa) facilitated the penetration of more rhodamine B into the dermis compared to classical ethosomes.^176^ Research indicates that HA can be included in topical skin drug delivery systems for the treatment of psoriasis and atopic dermatitis. Its mucoadhesive properties enable controlled drug release over time and absorption rate.^204^ The use of etosomes, which are based on propylene glycol coated with a gel made of HA with MW = 240 kDa, resulted in the delivery of 1% curcumin locally to the dermis and transdermally after 8 h *in vitro* on mouse skin at levels 1.4 and 1.6 times higher, respectively, compared to ethosomes without HA. Additionally, the delivery was 3.3 and 3.1 times higher compared to the curcumin solution alone.^90^

In the treatment of atopic dermatitis, polymeric nanoparticles coated with HA with a MW of 100 kDa have been shown to be effective in the *ex vivo* delivery of tacrolimus to rat skin, according to Zhuo et al.^91^ Other polymer nanoparticles coated with HA of the same molecular weight have also been used to deliver betamethasone valerate to *ex vivo* rat skin.^92^

Table 1 (items 34–52) presents the permeation effectiveness of APIs used with HA in TTDs.

## 5 Conclusion

The use of natural substances possessing properties that facilitate API permeation through the SC is undoubtedly the direction in which modern pharmaceuticals are heading.

The disorganizing effect on lamellar lipid fractions is considered the most crucial factor in achieving improved permeation. Urea is one such compound that can cause this effect. Terpenes are widely recognized as the most versatile permeation enhancers due to their molecular structure, which contains both polar and nonpolar groups. This structure enables terpenes to promote the permeation of both hydrophilic and lipophilic APIs. Additionally, HA possesses mucoadhesive properties that allow for controlled drug release over time and absorption rate.

API penetrations appear to be most effective when utilizing a compound formulation containing a mixture of permeation enhancers that complement each other, as they can interact with both the lipids and proteins of the SC, thereby creating transport pathways for both hydrophobic and hydrophilic APIs. This method also avoids the application of high concentrations of a single permeation enhancer, which could induce overly potent changes in the epidermis and cause skin irritation. Furthermore, this approach offers the possibility of effective use of both polar and nonpolar APIs.

We believe that proposed look on so diverse group of substances that contribute to transdermal delivery system offers new perspective for further study leading to discovery of new, natural permeation enhancers.

## Abbreviations

TDDs: transdermal delivery systems
SC: *stratum corneum*
CPEs: chemical permeation enhancers
API: active pharmaceutical ingredient
SPP: short periodicity phase
LPP: long periodicity phase
NMF: natural moisturizer factor
CER NS: nonhydroxy acyl sphingosine-type ceramide
E-PABA: ethyl-*p*-aminobenzoate
OA: oleic acid
HP-β-CD: hydroxy propyl-beta cyclodextrin
rhGH: recombinant human growth hormone
IU: isoprene unit
SER: skin electrical resistance
TEM: transmission electron microscope
CGMD: coarse-grained molecular dynamics
GC-MS: gas chromatography–mass spectrometry
TOF-SIMS: time-of-flight secondary ion mass spectrometry
SEM: scanning electron microscope
PASI: psoriatic area severity index
HA: hyaluronic acid
